# Deciphering the Mechanisms of Statin–Ezetimibe Drug Combinations Using Boolean Logical Modeling and Transcriptomic Data

**DOI:** 10.1002/psp4.70329

**Published:** 2026-09-01

**Authors:** Rui‐Sheng Wang, Matteo Pedrelli, Osman Ahmed, Garagnani Paolo, Paolo Parini, Joseph Loscalzo

**Affiliations:** ^1^ Department of Medicine Brigham and Women's Hospital Boston USA; ^2^ Cardio Metabolic Unit, Department of Medicine and Department of Laboratory Medicine Karolinska Institute Huddinge Sweden; ^3^ Medical Unit Endocrinology, Theme Inflammation and Ageing Karolinska University Hospital Stockholm Sweden; ^4^ Department of Biochemistry, College of Medicine and Health Sciences Arabian Gulf University Manama Kingdom of Bahrain; ^5^ Department of Experimental, Diagnostic and Specialty Medicine (DIMES) University of Bologna Bologna Italy

**Keywords:** Boolean logical modeling, drug combinations, ezetimibe, mechanisms of action, network biology, statins

## Abstract

Drug Combinations offer increased therapeutic efficacy and reduced toxicity compared with single agents. Understanding a drug combination's mechanisms of action (MoA) can provide important insights into therapeutic efficacy. The MoA of many FDA‐approved drugs, however, often remains unclear. To decipher the underlying molecular mechanisms of drugs used alone and in combination, we investigated the combination of a statin (atorvastatin or simvastatin) plus ezetimibe using drug‐treated RNA‐seq transcriptome data from the human hepatocyte‐like SOAT2‐only‐HepG2 cells and from liver biopsies of non‐obese normolipidemic patients with uncomplicated cholesterol gallstone disease in the Stockholm Study. We proposed a novel Boolean logical modeling framework to simulate the MoA of a drug combination using fourteen two‐variable Boolean models. Thereafter, a pattern matching approach was applied to associate drug‐induced differentially expressed genes with the idealized differential expression templates derived from Boolean models. We found 1560 and 565 genes differentially expressed in at least one treatment condition in SOAT2‐only‐HepG2 cells and liver biopsies, respectively. Our analysis revealed both expected and novel combinatorial modes of the statins and ezetimibe. We mapped the downstream genes of each combinatorial mode to the human protein–protein interactome and obtained underlying pathways, which are important for understanding the therapeutic effects of the drug combinations. Functional enrichment and disease‐association analyses of the downstream genes also provide critical insights into the additional therapeutic actions of the drugs. Our study demonstrates that drug‐induced transcriptomes, integrated with the human interactome, are informative in deciphering the MoA of drug combinations using Boolean logical modeling.

## Study Highlights

- What is the current knowledge on the topic?
  ○ Drug combinations such as statins plus ezetimibe are widely used to enhance lipid‐lowering efficacy and reduce adverse effects. Although the primary targets of these drugs are known, the molecular mechanisms underlying their combined actions remain unclear
- What question did this study address?
  ○ This study used Boolean logical modeling of drug‐induced transcriptomic profiles and the human protein–protein interactome to systematically infer the molecular mechanisms of action of a statin–ezetimibe combination.
- What does this study add to our knowledge?
  ○ This study revealed both expected and previously unrecognized combinatorial effects of statins and ezetimibe. Mapping downstream genes to the human interactome identified mechanistically interpretable subnetworks and pathways associated with specific modes of action.
- How might this change drug discovery, development, and/or therapeutics?
  ○ This framework provides a generalizable approach for mechanistic interpretation of drug combinations using transcriptomic and interactome data. It may support rational design and evaluation of combination therapies, improve translational interpretation of patient‐derived omics data, and help uncover additional therapeutic actions or system‐level effects relevant to clinical pharmacology.

## 1 Introduction

In many cases, taking more than one drug is necessary to cure a disease, alleviate symptoms, or manage a chronic condition. A drug combination refers to the use of two or more active pharmaceutical ingredients together to treat a medical condition. These drugs can be administered in a single dosage form or as separate medications taken together as part of a treatment plan [1]. Drug combinations can enhance therapeutic efficacy through additive or synergistic effects, reducing potential toxicity secondary to lower dosage. Indeed, combination therapies provide more efficient treatments for multiple diseases from hypertension to cancer [2, 3, 4].

A drug's mechanism of action (MoA) includes specific targets or pathways through which the compound produces its pharmacological effect in a specific cellular context. Elucidation of the MoA of a drug is critical to assess on‐target compound activity, rationalize phenotypic findings, and anticipate potential off‐target side effects [5]. Knowledge of the molecular mechanisms of existing combination therapies can provide clues to aid the discovery of new drug combinations and multi‐targeted agents [6]. However, elucidation of the MoA of a drug or drug combination is a challenging task in the drug discovery process that is only partially addressed by conventional experimental and computational strategies. For this reason, the functions of many FDA‐approved drugs remain unclear, especially when used in combination regimens.

A variety of methods have been proposed for predicting effective drug combinations [7, 8]. Most experimental approaches for MoA characterization rely on direct binding assays to putative targets, which are labor‐intensive and limited to the identification of high‐affinity binding targets [9, 10]. Chemo‐informatics methods are mostly designed to assess the MoA similarity of compounds or specific compound/target interactions by integrating structural and genomic information [11]. Computational methods have been developed to analyze the MoA of drugs based on drug‐induced omics data [12]. Network‐based methods perform integrative analyses over interacting gene sets and elucidate the therapeutic effect of drugs by modeling the action of drugs and diseases at a systems level. Therefore, network approaches can better explain the interplay between disease perturbations and drug targets, as well as how drug compounds induce favorable biological responses and/or adverse effects [5, 13, 14].

Although computational methods have been developed to analyze the mechanisms of action of single drugs, understanding the MoA of drug combinations is considerably more challenging as drug–drug interactions must also be taken into account. Boolean logical modeling has been widely used to simulate signal transduction networks and predict drug synergy [15, 16]. In this study, we propose a Boolean modeling framework to generate mechanistic hypotheses for drug combinations through analysis of drug‐induced transcriptomic perturbations. Statins, including atorvastatin and simvastatin, are 3‐hydroxy‐3‐methyl‐glutaryl‐coenzyme A (HMG‐CoA) reductase inhibitors commonly used to treat hypercholesterolemia by inhibiting endogenous cholesterol synthesis. Ezetimibe lowers cholesterol levels by blocking the Niemann‐Pick C1‐like 1 (NPC1L1) receptor, thereby reducing intestinal and biliary cholesterol absorption. Although the therapeutic efficacy of statin–ezetimibe combinations has been investigated experimentally [17, 18, 19], their underlying mechanisms of action have been barely explored. While one may simply assume the therapeutic effects simply reflect the additive inhibition of cholesterol synthesis by statins and cholesterol absorption by ezetimibe, the complex network of molecular interactions regulating cholesterol metabolism suggests a much more intricate biological response. We therefore hypothesized that statins, ezetimibe, and their combination induce distinct transcriptional programs. To test this hypothesis, we created fourteen two‐variable Boolean models to describe the combinatorial relationships of statins and ezetimibe. To evaluate the applicability of the proposed Boolean framework across different experimental settings, we applied it independently to two transcriptomic datasets: SOAT2‐only‐HepG2 cells treated with atorvastatin and ezetimibe, and liver biopsies from patients treated with simvastatin and ezetimibe. These datasets were analyzed independently using the same computational framework rather than for direct biological comparison.

## 2 Materials and Methods

Additional details of the methods are provided in the online only Supplement.

### 2.1 Drug‐Induced Transcriptome Datasets

The first drug‐induced RNA‐seq dataset was derived from human SOAT2‐only‐HepG2 cells—a unique human hepatocyte cell model that better simulates lipid and lipoprotein metabolism [20] (cultured in human sera as described in [19, 20, 21] and treated for 16 h with atorvastatin 5 umol/L, ezetimibe 25 umol/L, their combination, or vehicle). There were 6 replicates for each treatment, and, in total, there were 24 samples and 63,568 genomic features in this dataset. To ensure that the genes we investigated had meaningful changes, we used ANOVA to identify 1560 genes differentially expressed across the four conditions.

Another drug‐induced RNA‐seq dataset we utilized is obtained from liver biopsies of non‐obese normolipidemic patients with uncomplicated cholesterol gallstone disease in the Stockholm Study (a single‐blind, randomized trial) who received simvastatin 80 mg daily, ezetimibe 10 mg daily, alone or in combination, or placebo for four weeks prior to sample collection and liver biopsy [22]. In total, there were 27 samples and 58,381 genomic features. The PCA map (Figure S1) shows that the patient data are more heterogeneous than the cell data. We used ANOVA to identify 565 genes that were differentially expressed across the four conditions.

### 2.2 Boolean Models for the Combinatory Actions of Drugs

**FIGURE 1:**
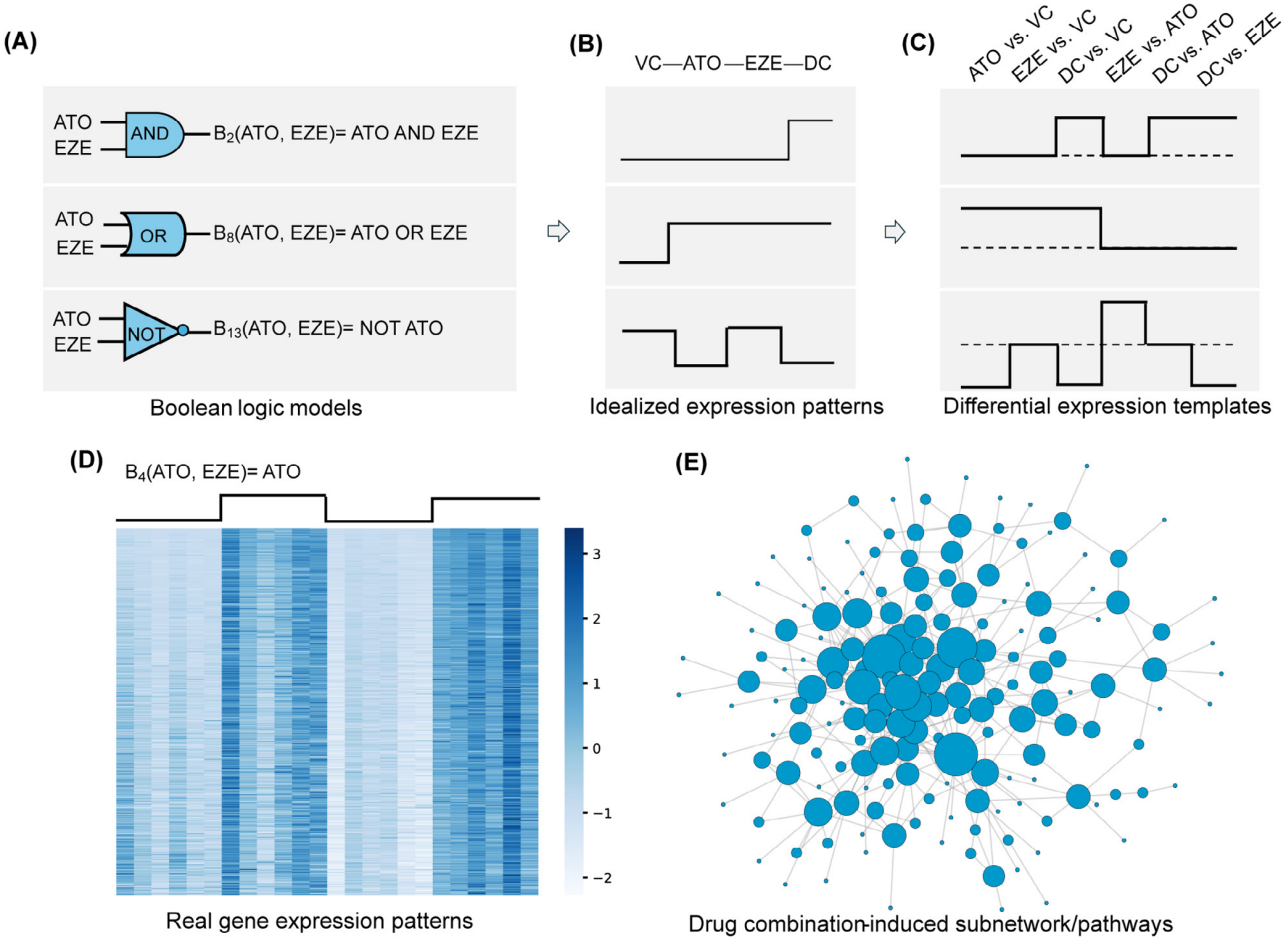
Boolean logical modeling framework for deciphering the MoA of drug combinations using drug‐induced transcriptomes. (A) The fourteen two‐variable Boolean logic functions represent the combinatorial modes of atorvastatin and ezetimibe. ATO and EZE are binary variables indicating the presence or absence of atorvastatin and ezetimibe treatment, respectively. (B) Idealized gene expression patterns across vehicle control (VC), atorvastatin (ATO), ezetimibe (EZE), and the atorvastatin‐ezetimibe drug combination (DC), as defined by the fourteen Boolean functons. (C) idealized differential gene expression patterns across the six pairwise comparisons among the four treatment conditions, corresponding to the fourteen combinatorial modes. (D) Oberseved gene expression patterns across the four treatment conditions corresponding to a combinatorial mode. (E) Protein interaction subnetworks induced by the downstream genes associated with the drug combination.

We adapted a Boolean framework [23] to model the combinatorial modes of action of drug combinations. We defined three binary variables: ATO, SIM, and EZE indicating atorvastatin, simvastatin, and ezetimibe treatments, respectively (Figure 1A). The full set of 16 Boolean functions defined by the two variables is summarized in Table S5 (Supplementary File 1) to describe the combinatorial modes of the statins and ezetimibe. Even though we use Boolean models, our method does not need to truncate gene expression profiles into binary values. Instead, we use Boolean models as idealized expression templates (Figure 1B).

### 2.3 Idealized Differential Expression Templates

To assign differentially expressed genes to the different combinatorial modes of action of a drug combination, we define idealized differential expression templates derived from the 16 Boolean models (Figure 1C). B_1_ and B_16_ represent the expression profiles of genes that do not change over different treatments and that have been filtered out during the ANOVA analysis. The remaining 14 Boolean models each define an idealized differential expression template through pairwise comparisons of two treatment conditions (Table S5, Supplementary File 1).

### 2.4 Pattern Matching

In order to associate differentially expressed genes with different regulatory action modes of the drug combination, we define an association metric between the differential expression pattern D*i* = (logFC_*i*,1_, logFC_*i*, 2_,…, logFC_*i, 6*_) of a gene *i* with the three‐valued differential expression template *T* = [*t*_1_, *t*_2_,…, *t*_6_] of a Boolean function B(ATO, EZE) (Supplemental File 1). LogFC denotes the log2‐transformed fold change in gene expression between two conditions, estimated using the R package Edge R. Each gene has 14 association scores representing its matching extent with the 14 regulatory modes of the drug combination and will be assigned to the one with maximum matching extent. The pattern matching approach takes the original differential expression profiles as input instead of truncating differential gene expression patterns into trinary values.

### 2.5 Human Protein–Protein Interactome

In our previous research, we have curated a high‐quality human protein–protein interactome consisting of 16,454 proteins and 233,957 physical protein–protein interactions (PPIs) directly from experimental high‐throughput interactome studies [24]. The inclusion of the most recent reference map of the human binary protein interactome (HuRI) [25] and Bioplex 3.0 [26] leads to the latest version of the human interactome consisting of 17,350 proteins and 350,950 interactions.

## 3 Results

### 3.1 Biologically Plausible Combinatorial Action Modes of Atorvastatin and Ezetimibe in SOAT2‐Only‐HepG2 Cells

**FIGURE 2:**
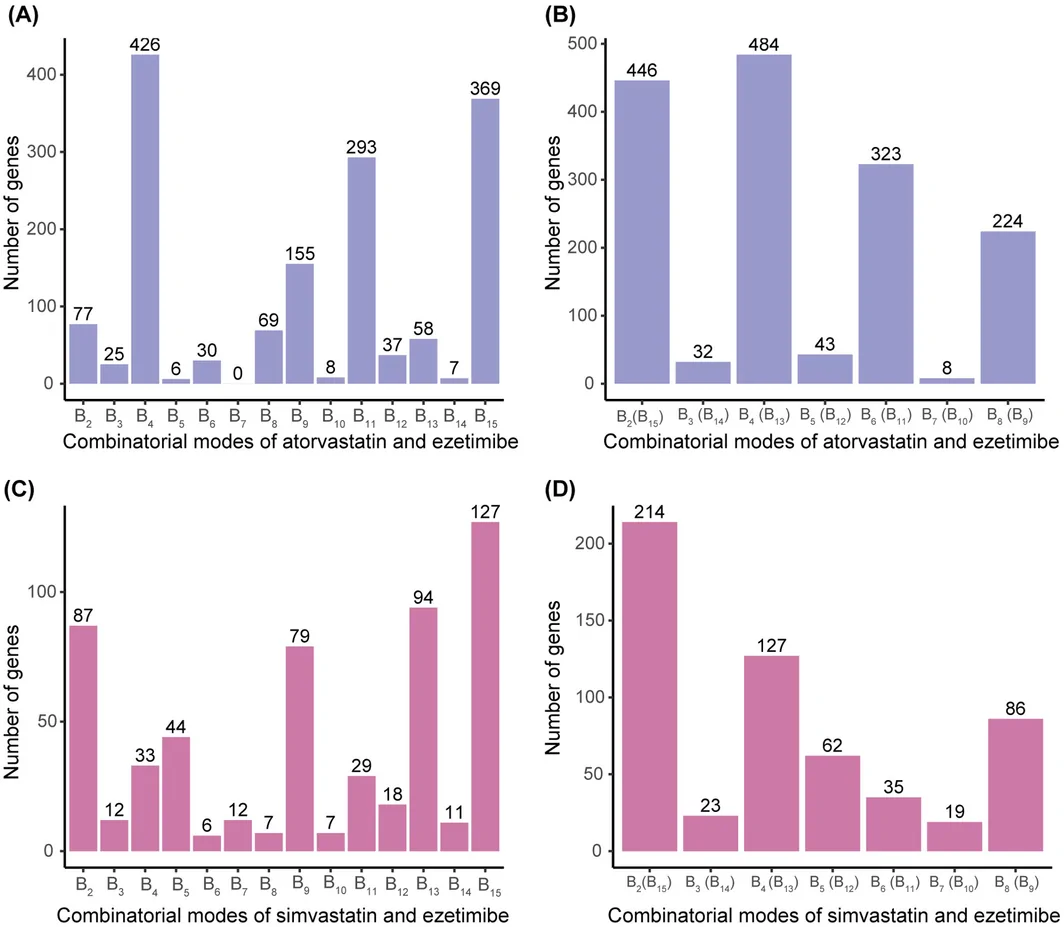
Distribution of genes assigned to different combinatorial modes for statin–ezetimibe drug combinations. (A) Distribution of genes across the fourteen combinatorial modes in SOAT2‐only‐HepG2 cells treated with atorvastatin and ezetimibe. (B) Distribution of genes in SOAT2‐only‐HepG2 cells after combining each combinatorial mode with its complementary (logical negation) mode (e.g., B_2_ with B_15_ and B_3_ with B_14_). (C) Distribution of genes across fourteen combinatorial modes in liver biopsies from patients treated with simvastatin and ezetimibe. (D) Distribution of genes in liver biopsies from patients after combining each combinatorial mode with its complementary mode.

Our first aim is to determine which of the theoretically possible combinatorial modes of atorvastatin and ezetimibe, modeled by the 14 Boolean functions B_2_ to B_15_, are experimentally supported by the drug‐induced transcriptome data from SOAT2‐only‐HepG2 cells. The distribution of genes in each combinatorial mode is provided in Figure 2A. Atorvastatin independently activates (upregulates) downstream genes (i.e., B_4_ = ATO), and atorvastatin and ezetimibe synergistically inhibit (downregulate) downstream genes (i.e., B_15_ = NOT (ATO AND EZE)), as the two most prevalent MoAs (426 and 369 genes, respectively). Other biologically plausible action modes include B_11_ = NOT EZE which indicates that genes are downregulated by ezetimibe independently, and B_9_ = NOT (ATO OR EZE), representing a condition in which genes are downregulated by either atorvastatin or ezetimibe, or both. The top seven prevalent combinatorial modes of atorvastatin and ezetimibe are summarized in Table 1. These action modes represent conditions in which atorvastatin and ezetimibe either independently or synergistically activate or inhibit downstream genes, which are biologically plausible.

**TABLE 1:** Prevalent and less possible combinatorial modes of atorvastatin and ezetimibe.

**Prevalent action modes**	**Implications**
B_4_(ATO, EZE) = ATO	Genes are upregulated by atorvastatin independently
B_11_(ATO, EZE) = NOT EZE	Genes are downregulated by ezetimibe independently
B_15_(ATO, EZE) = NOT (ATO AND EZE)	Genes are downregulated only by the combination of atorvastatin and ezetimibe
B_9_(ATO, EZE) = NOT (ATO OR EZE)	Genes are downregulated by either atorvastatin or ezetimibe, or both
B_2_(ATO, EZE) = ATO AND EZE	Genes are upregulated only by the combination of atorvastatin and ezetimibe
B_8_(ATO, EZE) = ATO or EZE	Genes are upregulated by either atorvastatin or ezetimibe, or both
B_13_(ATO, EZE) = NOT ATO	Genes are downregulated by atorvastatin independently
**Less possible action modes**	**Implications**
B_7_(ATO, EZE) = (ATO OR EZE) AND NOT (ATO AND EZE)	Genes are upregulated by either atorvastatin or ezetimibe, but not their combination
B_5_(ATO, EZE) = NOT ATO AND EZE	Genes are upregulated by ezetimibe when atorvastatin is not present
B_14_(ATO, EZE) = NOT ATO OR EZE	Genes are downregulated by atorvastatin when ezetimibe is not present
B_10_(ATO, EZE) = NOT (ATO OR EZE) OR (ATO AND EZE)	Genes are downregulated by either atorvastatin or ezetimibe, but not their combination
B_3_(ATO, EZE) = ATO AND NOT EZE	Genes are upregulated by atorvastatin when ezetimibe is not present
B_6_(ATO, EZE) = EZE	Genes are upregulated by ezetimibe independently
B_12_(ATO, EZE) = ATO OR NOT EZE	Genes are downregulated by ezetimibe when atorvastatin is not present

In contrast, some other combinatorial modes appear to be less prevalent (Table 1). For example, B_7_ = (ATO OR EZE) AND NOT (ATO AND EZE), representing conditions in which genes are upregulated by either atorvastatin or ezetimibe, but not their combination, has no assigned genes. B_10_ = NOT (ATO OR EZE) OR (ATO AND EZE), meaning that genes are downregulated by either atorvastatin or ezetimibe but not their combination, only has 8 associated genes. Other less prevalent combinatorial modes include B_5_ = NOT ATO AND EZE, denoting genes that are upregulated by ezetimibe when atorvastatin is not present, and B_14_ = NOT ATO OR EZE, indicating that genes are downregulated by atorvastatin when ezetimibe is not present. These modes are less possible as both atorvastatin and ezetimibe are cholesterol‐lowering medications used to treat high cholesterol levels. Surprisingly, B_6_ = EZE only has 30 genes. This outcome may reflect the expected lower hypocholesterolemic potency of this drug compared to statins, especially in cell culture systems.

Note that it is likely that the genes in complementary Boolean functions B_*i*_ and B_17‐*i*_ are regulated by atorvastatin and ezetimibe through the same signaling pathway. For example, for B_2_ and B_15_, atorvastatin and ezetimibe could synergistically regulate a mediator (e.g., a transcription factor or other regulator of gene expression), which activates the genes in B_2_ and inhibits the genes in B_15_, as likely results of lower intracellular cholesterol levels. To gain insight into the overall abundance of possible combinatorial regulatory mechanisms of the two drugs, we plot the cumulative number of genes in each combinatorial mode by merging the genes present in B_17‐*i*_ with those in B_*i*_, as shown in Figure 2B. We can see that the prevalent combinatorial modes are atorvastatin and ezetimibe's synergistic regulation (B_2_, B_15_), atorvastatin's independent regulation (B_4_ and B_13_), ezetimibe's independent regulation (B_6_ and B_11_), and atorvastatin and ezetimibe's disjunctive regulation (B_8_ and B_9_). In contrast, the exclusive regulatory modes (B_3_ and B_14_, B_5_ and B_15_) and the antagonistic regulatory modes (B_7_ and B_10_) of atorvastatin and ezetimibe have very few genes.

### 3.2 Prevalent Combinatorial Action Modes of Simvastatin and Ezetimibe in the Liver Biopsies

Next, we determined which of the theoretically possible combinatorial modes of simvastatin and ezetimibe are experimentally supported by the drug‐induced transcriptome data in the liver biopsies of patients with uncomplicated cholesterol gallstone disease in the Stockholm Study. Both simvastatin and atorvastatin are members of the statin class of drugs, used primarily to lower cholesterol and reduce the risk of heart disease by inhibiting HMG‐CoA reductase, the rate‐limiting enzyme in endogenous cholesterol synthesis, up‐regulating in turn low density lipoprotein (LDL) receptors. As shown in Figure S1, the liver biopsy transcriptomes exhibited substantially greater biological variability than the SOAT2‐only‐HepG2 cells, as expected because liver biopsies contain multiple cell types and patient‐to‐patient heterogeneity. Consequently, considerably fewer genes were identified as differentially expressed in the liver biopsy dataset. After we assigned the differentially expressed genes to different combinatorial modes of simvastatin and ezetimibe (Figure 2C), we found that some prevalent modes are conserved in liver biopsies vs. SOAT2‐only‐HepG2 cells, such as B_2_, B_4_, B_9_, B_11_, B_13,_ and B_15_. The greatest difference is in B_5_ which has only 6 out of 1560 genes in SOAT2‐only‐HepG2 cells but has 44 out of 565 genes in the liver biopsies. The difference may be a consequence of the fact that simvastatin and atorvastatin differ in their potency, dosing, and underlying metabolism, but also that liver biopsy data capture signals that do not originate exclusively from hepatocytes. When we accumulate the number of assigned genes by considering the complementation of Boolean functions (Figure 2D), the prevalence of all the combinatorial modes except for B_2_ (B_15_) was remarkably similar between SOAT2‐only‐HepG2 cells and liver biopsies. This finding suggests that the Boolean framework captures conserved patterns of transcriptional responses despite differences between the in vitro and in vivo experimental systems, the greater biological heterogeneity of patient liver biopsies, and the use of different statins.

**FIGURE 3:**
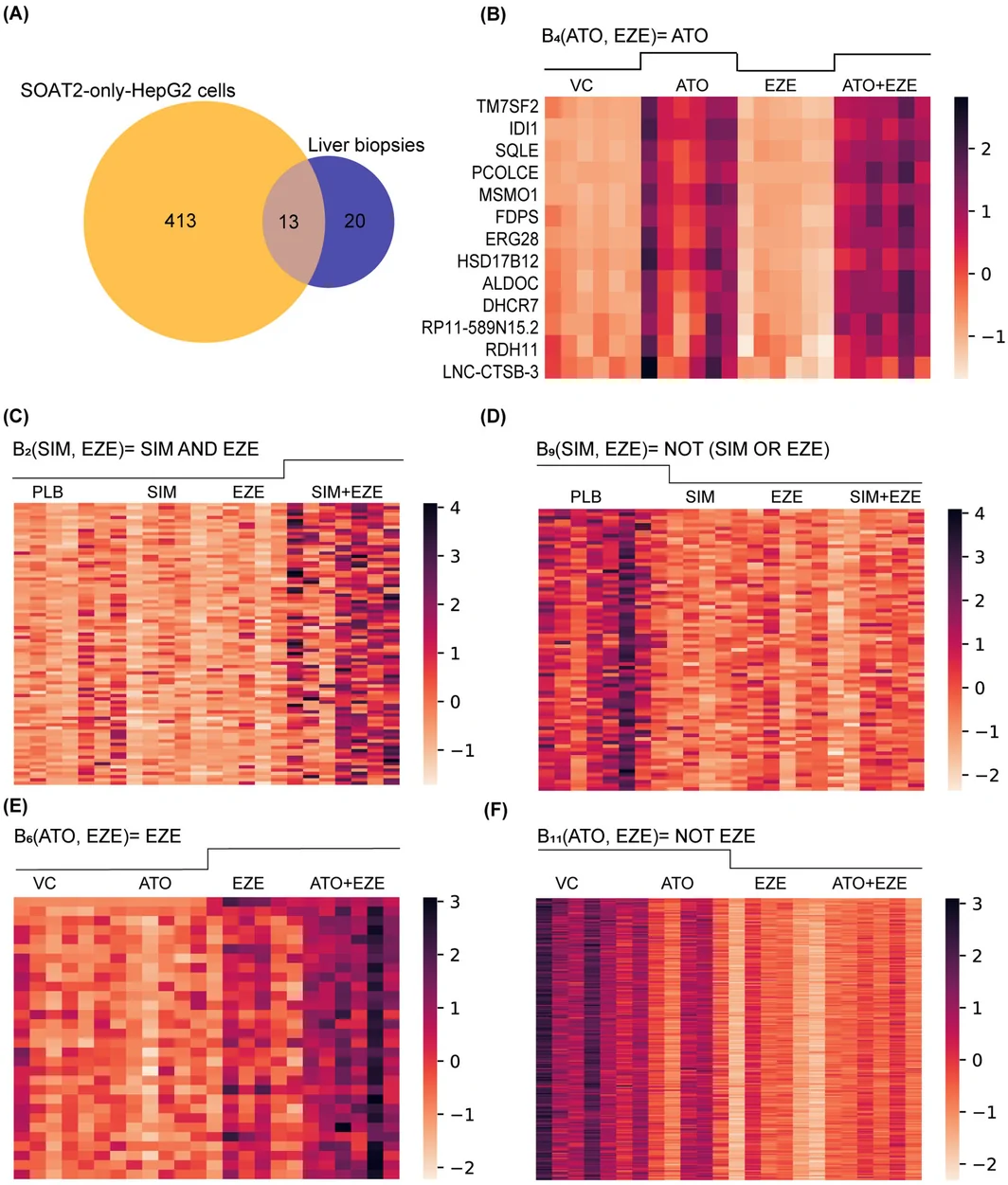
Observed gene expression patterns induced by representative combinatorial modes of atorvastatin/simvastatin and ezetimibe. (A) Overlap of genes assigned to B_4_(ATO, EZE) and B_4_(SIM, EZE). The overlap is statistically significant. (B) The 13 genes shared between B_4_(ATO, EZE) and B_4_(SIM, EZE). (C) Gene expression pattern induced by the synergistic mode of simvastatin and ezetimibe (B_2_(SIM, EZE)) in liver biopsies. (D) Gene expression pattern corresponding to B_9_(SIM, EZE) in liver biopsies. (E) Gene expression pattern upregulated by ezetimibe independently in SOAT2‐only‐HepG2 cells (B_6_(ATO, EZE)). (F) Gene expression pattern downregulated by ezetimibe independently in SOAT2‐only‐HepG2 cells (B_11_(ATO, EZE)).

We next compared the genes assigned to the same combinatorial modes in liver biopsies and SOAT2‐only‐HepG2 cells to identify conserved transcriptional responses across the two independent datasets. Interestingly, only genes in B_4_ showed significant overlap (*p* < 2.5E‐11 hypergeometric test, Figures 3A–3B), while other combinatorial modes had little or no overlapping genes. This limited overlap is likely attributable to the substantially smaller number of differentially expressed genes identified in the heterogeneous liver biopsy dataset, together with differences in biological context, experimental system, and the specific statins used. The 13 overlapping genes represent a conserved cholesterol‐regulatory program shared between atorvastatin and simvastatin, and the majority of these are involved in cholesterol biosynthesis [27, 28, 29, 30]. Representative gene expression patterns associated with selected combinatorial modes are shown in Figure 3C–F. Overall, gene expression patterns were more consistent and distinct in SOAT2‐only‐HepG2 cells than in liver biopsies, reflecting the greater molecular and cellular heterogeneity of the patient samples.

**FIGURE 4:**
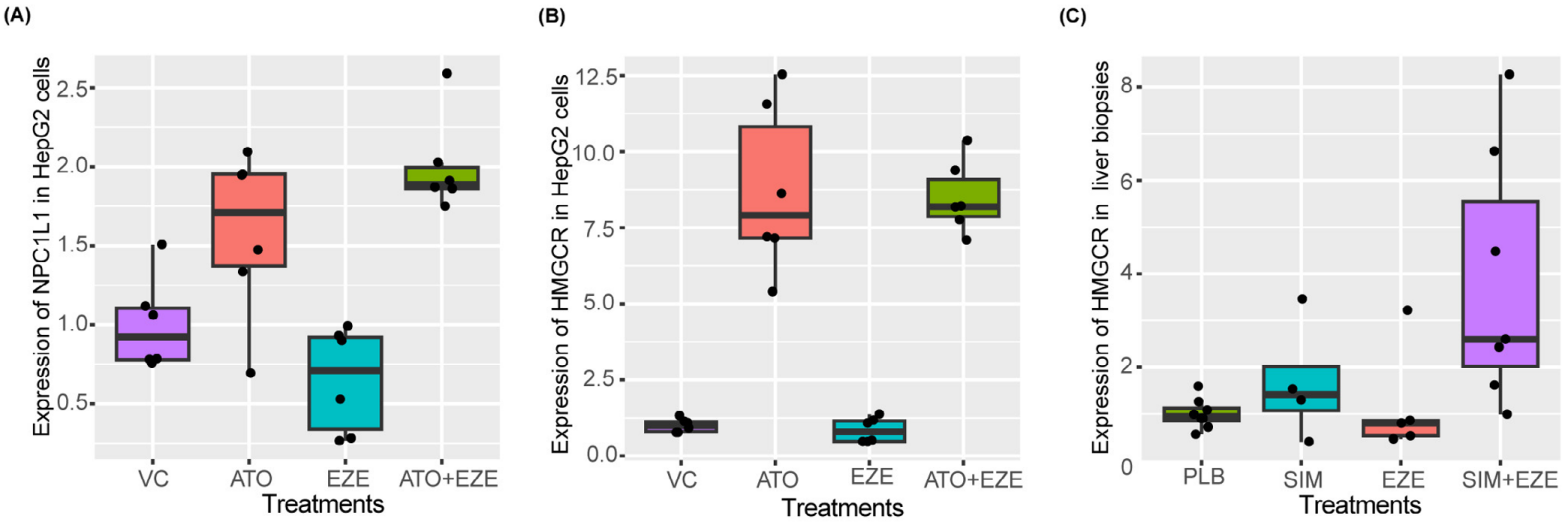
Normalized expression of target genes of statins and ezetimibe across four treatment conditions. (A) The expression of *NPC1L1*, the target gene of ezetimibe, in SOAT2‐only‐HepG2 cells. (B) The expression of *HMGCR*, the target gene of atorvastatin, in SOAT2‐only‐HepG2 cells. (C) The expression of *HMGCR* in liver biopsies from the patients. VC: Vehicle control; ATO: Atorvastatin; EZE: Ezetimibe; PLB: Placebo; SIM: Simvastatin.

According to DrugBank [31], atorvastatin has five target proteins: AHR, DPP4, HDAC2, HMGCR, and NR1I3. Simvastatin has two targets common to atorvastatin (HDAC2, HMGCR) with two additional targets (ITGAL, ITGB2). Ezetimibe has two target proteins: ANPEP and NPC1L1. NPC1L1 was assigned to B_4_(ATO, EZE) = ATO, indicating that atorvastatin induces the expression of NPC1L1 independent of ezetimibe (Figure 4A). HMGCR is a common target of atorvastatin and simvastatin. It was assigned to B_4_ in SOAT2‐only‐HepG2 cells which means the expression of HMGCR was induced by atorvastatin independent of ezetimibe (Figure 4B), but it was assigned to B_2_(SIM, EZE) = SIM AND EZE in liver biopsies (Figure 4C), which indicates that the elevated expression of HMGCR requires both simvastatin and ezetimibe. In fact, this difference is consistent with other studies showing that there is large pharmacodynamic variability between cell lines in cholesterol regulation [32] and that the signal needs profound “intracellular cholesterol depletion” in patients to emerge statistically from background noise.

### 3.3 Functional and Pathway Enrichment of Prevalent Combinatorial Modes of Atorvastatin/Simvastatin and Ezetimibe

**FIGURE 5:**
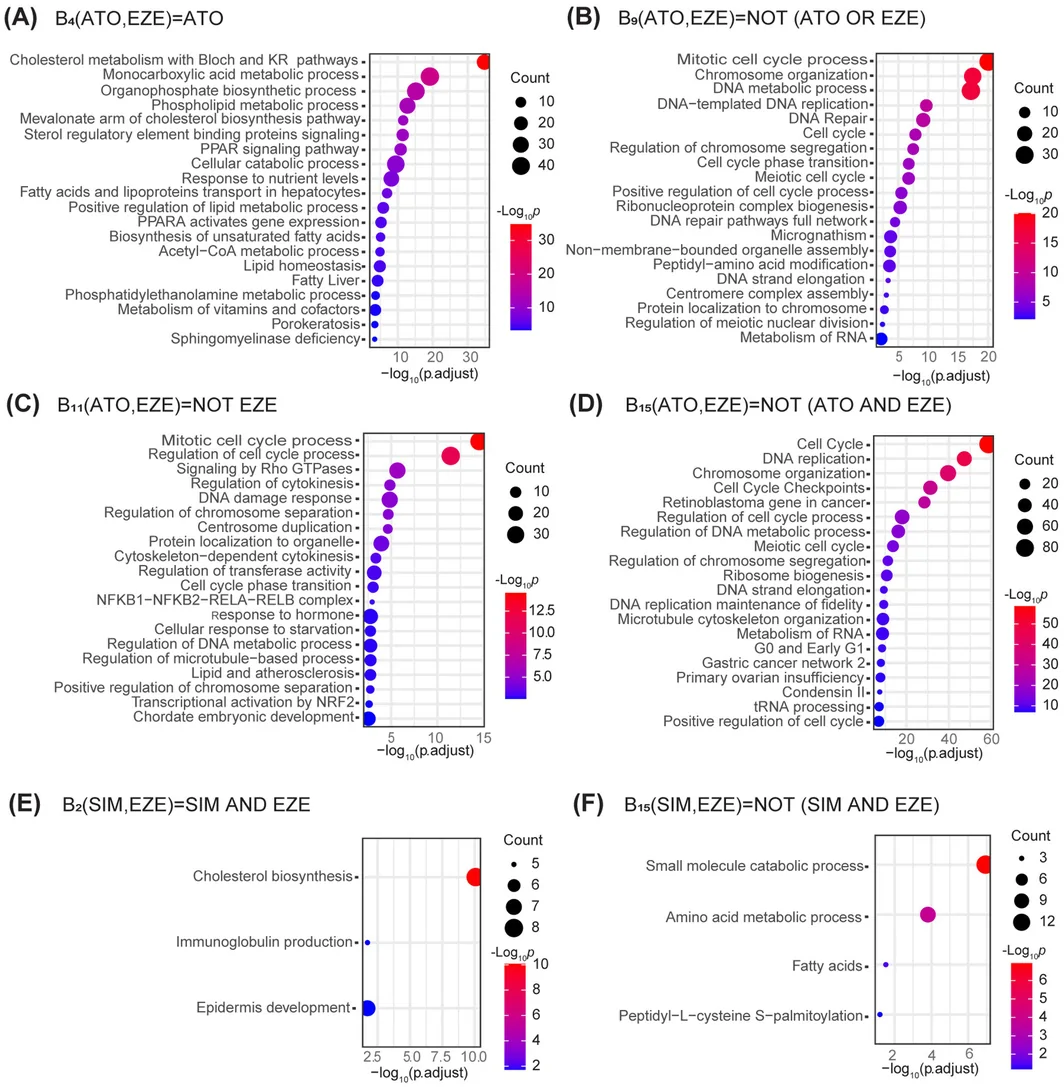
Pathway enrichment analysis of genes assigned to representative combinatorial modes of statins and ezetimibe. (A) Pathway enrichment of genes assigned to B_4_(ATO, EZE). (B) Pathway enrichment of genes assigned to B_9_(ATO, EZE). (C) Pathway enrichment of genes assigned to B_11_(ATO, EZE). (D) Pathway enrichment of genes assigned to B_15_(ATO, EZE). (E) Pathway enrichment of genes assigned to B_2_(SIM, EZE). (F) Functional enrichment of genes assigned to B_15_(SIM, EZE).

To examine whether specific combinatorial modes of the statins and ezetimibe tend to be involved in specific signaling pathways, we performed functional pathway analysis on the genes assigned in prevalent combinatorial modes using MetaScape [33] (Supplemental File 1). The top twenty function and pathway terms significantly enriched in the prevalent combinatorial modes of atorvastatin and ezetimibe are provided in Figure 5A–D. As expected, we observed that genes assigned to B_4_(ATO, EZE) = ATO are significantly enriched with pathways related to cholesterol biosynthesis and lipid metabolism. These pathways include cholesterol metabolism via Bloch and Kandutsch‐Russell pathways, the cholesterol biosynthesis pathway, lipid homeostasis, positive regulation of lipid metabolic processes, and fatty acids and lipid protein transport. This enrichment is consistent with the therapeutic effects of atorvastatin.

Genes assigned to B_9_ = NOT (ATO OR EZE), B_11_ = NOT EZE, and B_15_ = NOT (ATO AND EZE) are significantly enriched with many cell‐cycle related pathways, such as the mitotic cell cycle process, cell cycle phase transition, regulation of mitotic nuclear division, and DNA damage process. In addition to inhibiting cholesterol absorption and promoting a compensatory increase of cholesterol synthesis [34], previous research showed that ezetimibe prevents the progression of mitotic clonal expansion by arresting the cell cycle at the G0/G1 phase and inhibiting cell proliferation [35, 36]. It also has been demonstrated that atorvastatin can induce cell growth inhibition and G0/G1 phase cell cycle arrest, leading to senescence in hepatocellular carcinoma cells and pancreatic cancer cells [37, 38].

Owing to the limited number of genes assigned to the combinatorial modes of simvastatin and ezetimibe, only a few GO biological processes and pathways are significantly enriched in these groups. The top function and pathway terms significantly enriched in B_2_ and B_15_ are provided in Figure 5(E‐F). Cholesterol biosynthesis and small molecule catabolic processes are significantly enriched in the genes assigned to B_2_ = SIM AND EZE (upregulated by the combination of simvastatin and ezetimibe) and B_15_ = NOT (SIM AND EZE) (downregulated by the combination of simvastatin and ezetimibe), respectively.

### 3.4 Network Mapping and Disease Association of Prevalent Combinatorial Modes

The human protein–protein interactome consists of physical interactions between proteins, providing valuable insights into the functional connections of gene products in the cellular environment. We mapped the genes assigned to prevalent combinatorial modes to the consolidated human interactome and used the seed connector algorithm developed in our previous work [39] to derive the underlying subnetworks induced by these combinatorial modes. For example, the subnetwork for B_4_ = ATO, shown in Figure S2A, represents the signaling pathway and molecular mechanisms upregulated by atorvastatin independent of ezetimibe. To understand the additional indications and therapeutic mechanisms of the atorvastatin/simvastatin and ezetimibe combination, we examined whether the subnetwork is significantly enriched with disease‐related genes (Supplemental File 1). From Figure S2B we observe that the subnetwork is significantly enriched with lipid disorders and diseases caused by or associated with high cholesterol and triglycerides, such as hyperlipidemia, hypercholesterolemia, hypertriglyceridemia, fatty liver disease, myocardial ischemia, vascular disease, and cerebral artery occlusion. This finding indicates a strong association between these genes and pathways linked to lipid metabolism and cardiovascular health. The enrichment contributes to elucidating atorvastatin's MoA in modulating these pathways, while also enhancing our understanding of the drug's effects in preventing and managing conditions related to atherosclerotic cardiovascular disease (ASCVD).

**FIGURE 6:**
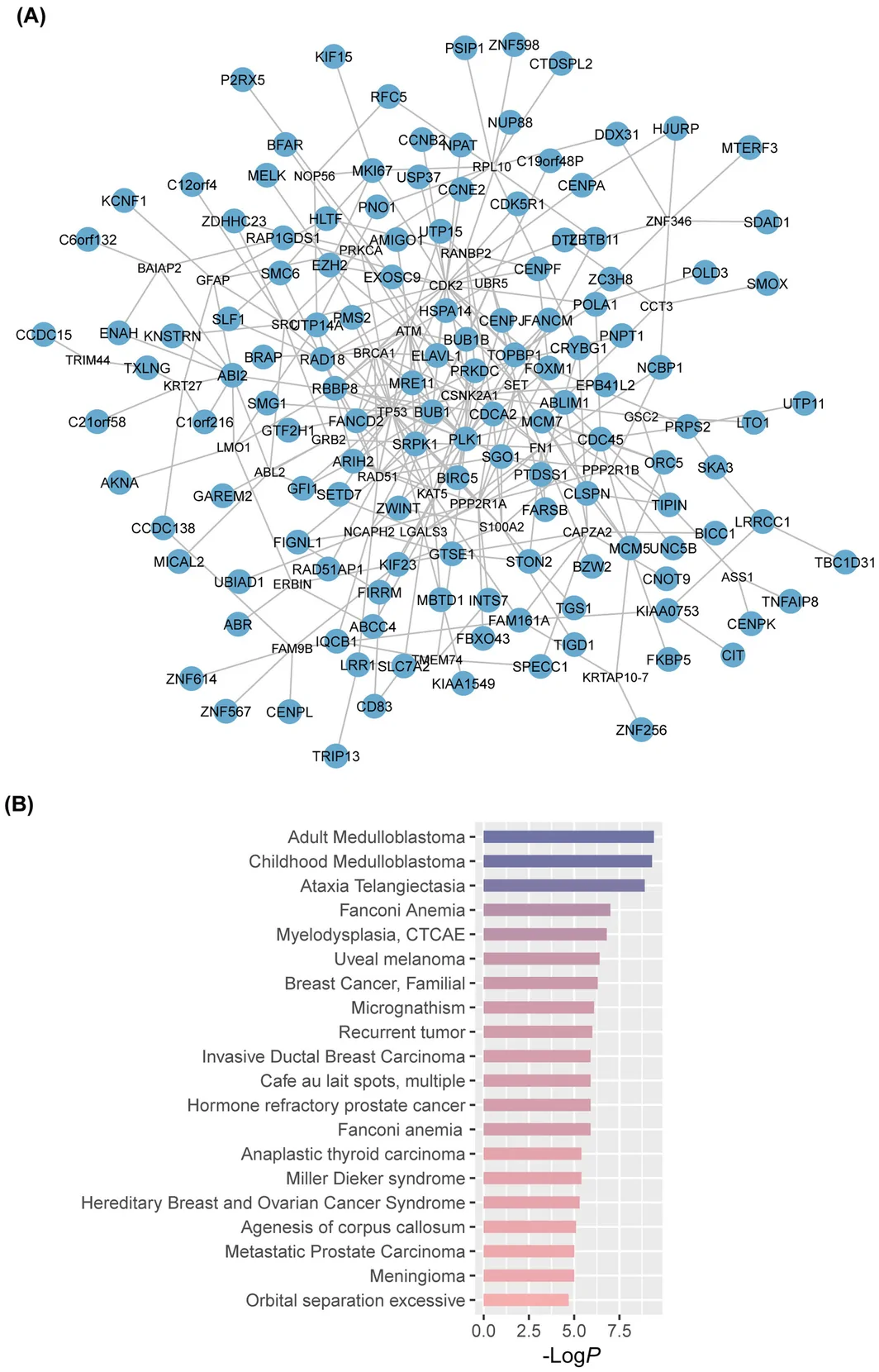
Protein interaction subnetwork and disease enrichment analysis of genes assigned to B_9_(ATO, EZE). (A) Protein–protein interaction subnetwork constructed by mapping the downstream genes assigned to B_9_(ATO, EZE) onto the human interactome. (B) Disease enrichment analysis of genes in the PPI subnetwork.

The subnetwork for B_9_ = NOT (ATO OR EZE) is shown in Figure 6A. This subnetwork represents the signaling pathway and molecular mechanisms downregulated by either atorvastatin or ezetimibe. The disease enrichment analysis shows that this subnetwork is significantly enriched with cancer‐related diseases, including medulloblastoma, breast cancer, and prostate cancer (Figure 6B).

## 4 Discussion

Understanding the MoA of drug combinations is essential for optimizing therapeutic efficacy, minimizing adverse effects, and guiding drug development. Although the pharmacological effects of statins and ezetimibe on cholesterol homeostasis are well established, systematically characterizing their genome‐wide combinatorial transcriptional responses remains challenging. In this study, we developed a Boolean logical modeling framework to classify transcriptomic responses into distinct combinatorial modes, thereby systematically organizing the molecular mechanisms underlying statin–ezetimibe combinations using transcriptomic data from human hepatocyte‐like SOAT2‐only‐HepG2 cells and liver biopsies from patients with uncomplicated cholesterol gallstone disease. By integrating these Boolean modes with the human protein–protein interactome, we further identified downstream subnetworks and biological pathways associated with each combinatorial mode. This framework provides a general strategy for interpreting transcriptomic responses to drug combinations and generating mechanistic hypotheses for further experimental investigation.

We identified several prevalent combinatorial modes of atorvastatin/simvastatin and ezetimibe, including B_4_(ATO, EZE) = ATO, representing genes independently upregulated by atorvastatin, a combinatorial mode that is most relevant to lipid disorders and ASCVD. These findings support the validity of our approach as statins were developed to decrease the atherogenic process by reducing the levels of cholesterol carried by apolipoprotein B‐containing lipoproteins (i.e., non‐HDL cholesterol). Because SREBP2, SREBP‐1c, and ChREBP are central transcriptional regulators of hepatocyte lipid and carbohydrate metabolism, our findings can be interpreted in relation to these established regulatory programs. The strongest concordance was observed for the SREBP2 axis. SREBP2 is activated by reduced cellular sterol availability and promotes transcription of genes involved in cholesterol biosynthesis and uptake. In agreement with the expected response to statin‐mediated inhibition of HMG‐CoA reductase, B_4_ contained SREBF2 together with several canonical SREBP2 target genes, including HMGCR, HMGCS1, LDLR, SQLE, and MVK. Thus, B_4_ likely represents a compensatory cholesterol‐depletion program induced primarily by atorvastatin. In addition, the observed atorvastatin‐associated increase in NPC1L1 is also compatible with this interpretation, as statin‐induced NPC1L1 upregulation has been reported in intestinal cells both in human and animal models and proposed as a compensatory response to cholesterol depletion within enterocytes through activation of SREBP2 and HNF‐4α [40]. This effect also appears to be mechanistically plausible in hepatocytes [19], where NPC1L1 is localized to the canalicular membrane and functions to reabsorb cholesterol from bile, counterbalancing cholesterol excretion.

In contrast, our data showed limited evidence for major engagement of SREBP‐1c‐ or ChREBP‐dominated transcriptional programs under the experimental conditions examined. SREBP‐1c primarily regulates fatty‐acid synthesis and lipogenic gene expression, whereas ChREBP, encoded by MLXIPL, is a carbohydrate‐responsive regulator of glycolytic and lipogenic transcription. Neither SREBF1 nor MLXIPL was differentially expressed across the four SOAT2‐only‐HepG2 treatment conditions, indicating that the dominant transcriptional response was more consistent with sterol depletion and SREBP2 activation than with broad activation of SREBP‐1c‐mediated de novo lipogenesis or ChREBP‐mediated carbohydrate‐responsive gene expression. This interpretation should be considered in light of the fact that these transcription factors are regulated not only at the transcript level but also through proteolytic processing, nuclear translocation, and transcriptional activity; therefore, transcriptomic data alone do not exclude post‐transcriptional changes in SREBP‐1c or ChREBP activity.

Another key novel finding is that B_4_(ATO, EZE) = ATO identified Alzheimer disease (AD)‐associated genes such as LRP8 and MTHFR. LRP8 (low‐density lipoprotein receptor‐related protein 8), a member of the LDL receptor family, mediates reelin signaling and interacts with apolipoprotein E (ApoE). Notably, the AD‐linked ApoE4 isoform traps LRP8 in endosomes, a defect that could potentially be mitigated by upregulating LRP8 to restore synaptic trafficking. Furthermore, LRP8 activation by reelin enhances NMDA receptor phosphorylation, counteracting amyloid‐beta (Aβ)‐induced synaptic suppression. This mechanism improves long‐term potentiation (LTP), a critical process for memory formation [41]. The current belief is that statins may be beneficial in AD by primarily influencing synaptic plasticity, cholesterol metabolism, and neuroprotection through mechanisms unrelated to LRP8. Hence, our findings can uncover genes with dual roles in lipid metabolism and neurodegeneration, offering new therapeutic targets for AD. MTHFR (methylenetetrahydrofolate reductase), which regulates homocysteine metabolism, harbors the C677T polymorphism that reduces enzyme activity, elevating homocysteine levels and promoting oxidative stress, DNA damage, and vascular dysfunction—mechanisms implicated in AD pathogenesis, with meta‐analyses confirming its increased AD risk (particularly in Asian populations) and elevated homocysteine correlating with gray matter atrophy in AD‐vulnerable regions like the hippocampus [42, 43]. As for LRP8, there were no data available as to whether statins upregulate the expression of *MTHFR* in brain and in the central nervous system (CNS), suggesting another interesting topic for further investigations.

Our approach also advocates for possible uses of atorvastatin or ezetimibe in reduction of diseases beyond those caused by atherosclerosis. The subnetwork included by B_9_(ATO, EZE) = NOT (ATO OR EZE) identified genes involved in oncogenesis, in accordance with recent evidence suggesting that cholesterol is a key regulator of cancer by influencing cell signaling events central to the hallmarks of malignant transformation [44]. Numerous studies have demonstrated the functional importance of cholesterol metabolism in tumorigenesis, cancer progression, and metastasis [45, 46]. Cholesterol exerts regulatory effects on the immune response, ferroptosis, autophagy, cell stemness, and the DNA damage response, which all contribute to cancer cell survival and proliferation. These findings underscore the potential impact of cholesterol‐modulating therapies, such as atorvastatin and ezetimibe, in influencing cancer pathways and point to a potentially broader role for lipid metabolism in oncology. Further investigation into this subnetwork could elucidate specific mechanisms by which lipid‐lowering therapies may mitigate cancer risks or progression.

This Boolean modeling framework formulates the coordinated (synergistic) MoA of drug combinations, which is consistent with our pursuit of the beneficial effects of drug combination. The limitation represented by using only transcriptomics in depicting the MoA can be mitigated by the use of pathway enrichment analysis and mapping the associated genes to the human protein–protein interactome to improve the ability to identify the underlying targets and pathways that the drug combination may alter. Our approach may also suggest unexpected and/or apparently inexplicable effects of drug combination, as by the B_7_ and the B_10_ modes, in which the concordant effects of either drug given alone are lost when given in combination, an outcome that would not be identified if we only consider the MoA for which the single agents have been designed. However, although less likely, different “off target” effects of the single agents may be divergent or attenuated when the drugs are given in combination. No genes were assigned to B_7_ and very few to B_10_. The first and second modes suggest that two agents discordantly upregulate and downregulate genes, respectively.

Based on our analysis of the human study data, we identified RGN and NCOA7‐AS1 among the seven genes assigned to the B_10_ model. This is an interesting finding, since genes play crucial roles in cellular defense mechanisms against oxidative stress [47], and since their combined action potentially enhances cellular protection against oxidative damage. Both genes are not known as direct targets of either statins or ezetimibe, and the fact that the combination of simvastatin and ezetimibe seems to blunt their downregulation occurring when patients receive those drugs as monotherapy, would suggest that combination therapy may have clear advantages not only regarding the lipid‐lowering activity but also regarding oxidative stress. Accordingly, CKD patients treated with ezetimibe/simvastatin combination showed more pronounced improvements in oxidative stress parameters compared to monotherapy [48]. Similarly, a randomized controlled trial comparing ezetimibe/simvastatin combination with rosuvastatin monotherapy in patients with diabetic polyneuropathy found that both treatments significantly reduced lipid peroxidation levels compared to placebo, with the combination therapy showing particular efficacy in reducing oxidative damage marker [49].

Our study has limitations. First, the Boolean logical framework is intended to classify transcriptomic response patterns rather than model the biochemical kinetics or dose–response relationships of drug action. Instead, Boolean logic serves as an abstraction of the observed transcriptional responses by categorizing genes according to significant induction or repression across different treatment conditions. Therefore, the Boolean combinatorial modes summarize qualitative patterns of gene regulation, whereas the underlying pharmacological processes remain continuous and dose dependent. Second, the transcriptomic data analyzed in this study were generated under a single exposure condition. Since drug‐induced transcriptional responses are influenced by drug concentration, treatment duration, and experimental context, different exposure conditions may alter the assignment of genes to specific combinatorial modes. Therefore, the combinatorial modes identified here represent the regulatory landscape under the experimental conditions examined rather than invariant properties of the drug combinations. Future studies incorporating dose–response and time‐course transcriptomic datasets will enable characterization of how combinatorial regulatory modes evolve across exposure conditions and further extend the applicability of this framework to other drug combinations and biological contexts. Finally, the two transcriptomic datasets analyzed in this study differed in experimental system, biological context, and statin treatment. Atorvastatin was selected for the SOAT2‐only‐HepG2 experiments because simvastatin is a lactone prodrug requiring conversion to its active hydroxy‐acid form [50], which could introduce an additional activation‐dependent variable in vitro; nevertheless, the use of different statins limits direct biological comparison with the simvastatin‐treated liver biopsy dataset. Consequently, differences between individual Boolean modes identified in the two datasets cannot be attributed to any single factor, such as the experimental model, biological variability, or the specific statin used. Future studies using matched experimental systems or standardized treatment conditions will be valuable for disentangling the relative contributions of these factors.

In conclusion, this study demonstrates that Boolean logical modeling provides an interpretable framework for organizing complex transcriptomic responses to drug combinations into biologically meaningful combinatorial modes. Rather than modeling the biochemical kinetics of drug action, the framework captures qualitative patterns of gene regulation and links them to downstream interaction networks and biological pathways. As transcriptomic datasets for combination therapies continue to expand, this approach has the potential to facilitate mechanistic interpretation of drug combinations, generate testable biological hypotheses, and support the rational design and evaluation of combination therapies in complex diseases.

## Author Contributions

R.‐S.W. designed the research; R.‐S.W. and M.P. performed the research; R.‐S.W, M.P., P.P., J.L. wrote the manuscript; R.‐S.W, M.P., P.P. O.A., G.P., J.L. analyzed the data; R.‐S.W., M.P., and P.P. contributed new reagents/analytical tools.

## Funding

The authors would like to acknowledge the support of their studies from NIH grants (HL155107, HL166137, and HG007691 to JL); from American Heart Association grants (AHA957729 and AHAA24 MERIT1185447 to JL; AHA25GWTGDRASp1454054 to RSW); and from EU HorizonHealth 2021 grant (101057619 to JL and RSW). To PP: Swedish Heart‐Lung Foundation (grant nr: 20210622; 20200668; 20190544), Swedish Research Council (grant nr: 201602992), and the financial resources provided from the Region Stockholm City Council, the Karolinska Institutet.

## Conflicts of Interest

The authors declare no conflicts of interest.

## Supporting information


**Table S1:** Within‐group sample correlation over all genes in SOAT2‐only‐HepG2 cells under four treatment conditions. VC: Vehicle control; ATO: atorvastatin; EZE: ezetimibe.
**Table S2:** Within‐group sample correlation over all genes in liver biopsies of patients given placebo treatment. PLA: placebo.
**Table S3:** Within‐group sample correlation over all genes in liver biopsies of patients given drug treatments. SIM: simvastatin; EZE: ezetimibe.
**Table S4:** Within‐group sample correlation over all genes in liver biopsies of patients given the combination of simvastatin and ezetimibe treatment. SIM: simvastatin; EZE: ezetimibe.
**Figure S1:** PCA mappings of samples from human SOAT2‐only‐HepG2 cells and liver biopsies. VC: Vehicle control; ATO: atorvastatin; EZE: ezetimibe. PLA: placebo. SIM: simvastatin; EZE: ezetimibe.
**Figure S2:** Network mapping (A) of genes assigned to B4(ATO, EZE) = ATO and disease enrichment analysis (B).
**Table S5:** Boolean functions modeling the combinatorial modes of atorvastatin and ezetimibe and the idealized gene expression patterns. In the case of simvastatin and ezetimibe, ATO is replaced with SIM.
**Table S6:** Boolean functions modeling the combinatorial modes of atorvastatin and ezetimibe and the idealized differential gene expression templates. In the case of simvastatin and ezetimibe, ATO is replaced with SIM, and VC is replaced with PLB (placebo).

**Data S1:** Supporting Information

## Data Availability

The RNA‐seq data generated from SOAT2‐only‐HepG2 cells are part of a separate study currently under review and will be made publically available upon publication of that study. The liver biopsy transcriptomic data are subject to data protection restrictions and access may be granted with approval from the relevant ethics committee. The code used for data analysis has been deposited in GitHub (https://github.com/bwh784/BooleanModeling).

## References

[1] X. Sun , S. Vilar , and N. P. Tatonetti , “High‐Throughput Methods for Combinatorial Drug Discovery,” Science Translational Medicine 5 (2023): 205rv201. 10.1126/scitranslmed.3006667 24089409

[2] G. Mancia , F. Rea , G. Corrao , and G. Grassi , “Two‐Drug Combinations as First‐Step Antihypertensive Treatment,” Circulation Research 124 (2019): 1113–1123. 30920930 10.1161/CIRCRESAHA.118.313294

[3] P. Jaaks , E. A. Coker , D. J. Vis , et al., “Effective Drug Combinations in Breast, Colon and Pancreatic Cancer Cells,” Nature 603 (2022): 166–173. 35197630 10.1038/s41586-022-04437-2 PMC8891012

[4] W. Zheng , W. Sun , and A. Simeonov , “Drug Repurposing Screens and Synergistic Drug‐Combinations for Infectious Diseases,” British Journal of Pharmacology 175, no. 2 (2018): 181–191. 28685814 10.1111/bph.13895 PMC5758396

[5] J. H. Woo , Y. Shimoni , W. S. Yang , et al., “Elucidating Compound Mechanism of Action by Network Perturbation Analysis,” Cell 162, no. 2 (2015): 441–451. 26186195 10.1016/j.cell.2015.05.056 PMC4506491

[6] J. Jia , F. Zhu , X. Ma , Z. W. Cao , Y. X. Li , and Y. Z. Chen , “Mechanisms of Drug Combinations: Interaction and Network Perspectives,” Nature Reviews Drug Discovery 8 (2009): 111–128. 19180105 10.1038/nrd2683

[7] F. Cheng , I. A. Kovács , and A.‐L. Barabási , “Network‐Based Prediction of Drug Combinations,” Nature Communications 10 (2019): 1197. 10.1038/s41467-019-09186-x PMC6416394 30867426

[8] B. G. Paltun , S. Kaski , and H. Mamitsuka , “Machine Learning Approaches for Drug Combination Therapies,” Briefings in Bioinformatics 22, no. 6 (2021): bbab293. 34368832 10.1093/bib/bbab293 PMC8574999

[9] J. G. C. van Hasselt and R. Iyengar , “Systems Pharmacology: Defining the Interactions of Drug Combinations,” Annual Review of Pharmacology and Toxicology 59 (2019): 21–40. 10.1146/annurev-pharmtox-010818-021511 30260737

[10] A. Ling and R. S. Huang , “Computationally Predicting Clinical Drug Combination Efficacy With Cancer Cell Line Screens and Independent Drug Action,” Nature Communications 11 (2020): 5848. 10.1038/s41467-020-19563-6 PMC7673995 33203866

[11] Y. Yamanishi , M. Araki , A. Gutteridge , W. Honda , and M. Minoru Kanehisa , “Prediction of Drug–Target Interaction Networks From the Integration of Chemical and Genomic Space,” Bioinformatics 24, no. 13 (2008): i232–i240. 18586719 10.1093/bioinformatics/btn162 PMC2718640

[12] M.‐A. Trapotsi , L. Hosseini‐Gerami , and A. Bender , “Computational Analyses of Mechanism of Action (MoA): Data, Methods and Integration,” RSC Chemical Biology 3, no. 2 (2022): 170–200. 35360890 10.1039/d1cb00069a PMC8827085

[13] F. Iorio , J. Saez‐Rodriguez , and D. di Bernardo , “Network Based Elucidation of Drug Response: From Modulators to Targets,” BMC Systems Biology 7 (2013): 139. 24330611 10.1186/1752-0509-7-139 PMC3878740

[14] T. J. Rintala , A. Ghosh , and V. Fortino , “Network Approaches for Modeling the Effect of Drugs and Diseases,” Briefings in Bioinformatics 23, no. 4 (2022): bbac229. 35704883 10.1093/bib/bbac229 PMC9294412

[15] R. S. Wang , A. Saadatpour , and R. Albert , “Boolean Modeling in Systems Biology: An Overview of Methodology and Applications,” Physical Biology 9, no. 5 (2012): 055001. 23011283 10.1088/1478-3975/9/5/055001

[16] K. Taoma , M. Ruengjitchatchawalya , M. Liangruksa , and T. Laomettachit , “Boolean Modeling of Breast Cancer Signaling Pathways Uncovers Mechanisms of Drug Synergy,” PLoS One 19, no. 2 (2024): e0298788. 38394152 10.1371/journal.pone.0298788 PMC10889607

[17] C. M. Ballantyne , J. Houri , A. Notarbartolo , et al., “Effect of Ezetimibe Coadministered With Atorvastatin in 628 Patients With Primary Hypercholesterolemia a Prospective, Randomized, Double‐Blind Trial,” Circulation 107 (2003): 2409–2415. 12719279 10.1161/01.CIR.0000068312.21969.C8

[18] C. Ai , S. Zhang , Q. He , and J. Shi , “Comparing the Combination Therapy of Ezetimibe and Atorvastatin With Atorvastatin Monotherapy for Regulating Blood Lipids: A Systematic Review and Meta‐Analyse,” Lipids in Health and Disease 17, no. 1 (2018): 239. 30326894 10.1186/s12944-018-0880-8 PMC6192348

[19] O. Ahmed , K. Littmann , U. Gustafsson , et al., “Ezetimibe in Combination With Simvastatin Reduces Remnant Cholesterol Without Affecting Biliary Lipid Concentrations in Gallstone Patients,” Journal of the American Heart Association 7, no. 24 (2018): e009876. 30561264 10.1161/JAHA.118.009876 PMC6405603

[20] C. Pramfalk , T. Jakobsson , C. R. C. Verzijl , et al., “Generation of New Hepatocyte‐Like in Vitro Models Better Resembling Human Lipid Metabolism,” Biochimica et Biophysica Acta ‐ Molecular and Cell Biology of Lipids 1865, no. 6 (2020): 158659. 32058035 10.1016/j.bbalip.2020.158659

[21] C. Pramfalk , L. Larsson , J. Härdfeldt , M. Eriksson , and P. Parini , “Culturing of HepG2 Cells With Human Serum Improve Their Functionality and Suitability in Studies of Lipid Metabolism,” Biochimica et Biophysica Acta 1861, no. 1 (2016): 51–59. 26515253 10.1016/j.bbalip.2015.10.008

[22] C. P. Cannon , M. A. Blazing , R. P. Giugliano , et al., “Ezetimibe Added to Statin Therapy After Acute Coronary Syndromes,” New England Journal of Medicine 372, no. 25 (2015): 2387–2397, 10.1056/NEJMoa1410489. 26039521

[23] S. Pandey , R. S. Wang , L. Wilson , et al., “Boolean Modeling of Transcriptome Data Reveals Novel Modes of Heterotrimeric G‐Protein Action,” Molecular Systems Biology 6 (2010): 372. 20531402 10.1038/msb.2010.28 PMC2913393

[24] R. S. Wang and J. Loscalzo , “Network Module‐Based Drug Repositioning for Pulmonary Arterial Hypertension,” CPT: Pharmacometrics & Systems Pharmacology 10, no. 9 (2021): 994–1005. 34132494 10.1002/psp4.12670 PMC8452304

[25] K. Luck , D.‐K. Kim , L. Lambourne , et al., “A Reference Map of the Human Binary Protein Interactome,” Nature 580 (2020): 402–408. 32296183 10.1038/s41586-020-2188-x PMC7169983

[26] E. L. Huttlin , R. J. Bruckner , J. Navarrete‐Perea , et al., “Dual Proteome‐Scale Networks Reveal Cell‐Specific Remodeling of the Human Interactome,” Cell 184, no. 11 (2021): 3022–3040.e28. 33961781 10.1016/j.cell.2021.04.011 PMC8165030

[27] A. C. Ramos‐Valdivia , R. van der Heijden , and R. Robert Verpoorte , “Isopentenyl Diphosphate Isomerase: A Core Enzyme in Isoprenoid Biosynthesis. A Review of Its Biochemistry and Function,” Natural Product Reports 14, no. 6 (1997): 591–603. 9418296 10.1039/np9971400591

[28] H. Yoshioka , H. W. Coates , N. K. Chua , Y. Hashimoto , A. J. Brown , and K. Ohgane , “A Key Mammalian Cholesterol Synthesis Enzyme, Squalene Monooxygenase, Is Allosterically Stabilized by Its Substrate,” Proc Natl Acad Sci USA 117, no. 13 (2020): 7150–7158. 32170014 10.1073/pnas.1915923117 PMC7132291

[29] I. M. Capell‐Hattam , N. M. Fenton , H. W. Coates , L. J. Sharpe , and A. J. Brown , “The Non Catalytic Protein ERG28 Has a Functional Role in Cholesterol Synthesis and Is Coregulated Transcriptionally,” Journal of Lipid Research 63, no. 12 (2022): 100295. 36216146 10.1016/j.jlr.2022.100295 PMC9730225

[30] A. V. Prabhu , W. Luu , D. Li , L. J. Sharpe , and A. J. Brown , “DHCR7: A Vital Enzyme Switch Between Cholesterol and Vitamin D Production,” Progress in Lipid Research 64 (2016): 138–151. 27697512 10.1016/j.plipres.2016.09.003

[31] C. Knox , M. Wilson , C. M. Klinger , et al., “DrugBank 6.0: The DrugBank Knowledgebase for 2024,” Nucleic Acids Research 52, no. D1 (2024): D1265–D1275. 37953279 10.1093/nar/gkad976 PMC10767804

[32] P. Blattmann , D. Henriques , M. Zimmermann , et al., “Systems Pharmacology Dissection of Cholesterol Regulation Reveals Determinants of Large Pharmacodynamic Variability Between Cell Lines,” Cell Systems 5 (2017): 604–619. 29226804 10.1016/j.cels.2017.11.002 PMC5747350

[33] B. Zhou , L. Pache , M. Chang , et al., Metascape provides a biologist‐oriented resource for the analysis of systems‐level datasets. 10.1038/s41467-019-09234-6 PMC6447622 30944313

[34] T. Sudhop , D. Lütjohann , A. Kodal , et al., “Inhibition of Intestinal Cholesterol Absorption by Ezetimibe in Humans,” Circulation 106, no. 15 (2002): 1943–1948. 12370217 10.1161/01.cir.0000034044.95911.dc

[35] Y. S. Lee , J. S. Park , D. H. Lee , J. Han , and S. H. Bae , “Ezetimibe Ameliorates Lipid Accumulation During Adipogenesis by Regulating the AMPK‐mTORC1 Pathway,” FASEB Journal 34, no. 1 (2020): 898–911. 31914598 10.1096/fj.201901569R

[36] L. Qin , Y.‐B. Yang , Y.‐X. Yang , et al., “Inhibition of Smooth Muscle Cell Proliferation by Ezetimibe via the Cyclin D1‐MAPK Pathway,” Journal of Pharmacological Sciences 125, no. 3 (2014): 283–291. 25048018 10.1254/jphs.13239fp

[37] S.‐T. Wang , S.‐W. Huang , K.‐T. Liu , T.‐Y. Lee , J.‐J. Shieh , and C.‐Y. Wu , “Atorvastatin‐Induced Senescence of Hepatocellular Carcinoma Is Mediated by Downregulation of hTERT Through the Suppression of the IL‐6/STAT3 Pathway,” Cell Death Discov 6 (2020): 17. 32257389 10.1038/s41420-020-0252-9 PMC7105491

[38] S. Cai , Q. Chen , Y. Xu , Q. Zhuang , and S. Ji , “Atorvastatin Inhibits Pancreatic Cancer Cells Proliferation and Invasion Likely by Suppressing Neurotrophin Receptor Signaling,” Translational Cancer Research 9, no. 3 (2020): 1439–1447. 35117491 10.21037/tcr.2020.01.27 PMC8798715

[39] R. S. Wang and J. Loscalzo , “Network‐Based Disease Module Discovery by a Novel Seed Connector Algorithm With Pathobiological Implications,” Journal of Molecular Biology 430, no. 18 Pt A (2018): 2939–2950. 29791871 10.1016/j.jmb.2018.05.016 PMC6097931

[40] A. J. Tremblay , B. Lamarche , V. Lemelin , et al., “Atorvastatin Increases Intestinal Expression of NPC1L1 in Hyperlipidemic Men,” Journal of Lipid Research 52, no. 3 (2011): 558–565. 21123766 10.1194/jlr.M011080 PMC3035692

[41] D. Passarella , S. Ciampi , V. D. Liberto , et al., “Low‐Density Lipoprotein Receptor‐Related Protein 8 at the Crossroad Between Cancer and Neurodegeneration,” International Journal of Molecular Sciences 23, no. 16 (2022): 8921. 36012187 10.3390/ijms23168921 PMC9408729

[42] Y. Hua , H. Zhao , Y. Kong , and M. Ye , “Association Between the MTHFR Gene and Alzheimer's Disease: A Meta‐Analysis,” International Journal of Neuroscience 121, no. 8 (2011): 462–471. 21663380 10.3109/00207454.2011.578778

[43] Y. Jiang , X. Xiao , Y. Wen , et al., “Genetic Effect of MTHFR C677T, A1298C, and A1793G Polymorphisms on the Age at Onset, Plasma Homocysteine, and White Matter Lesions in Alzheimer's Disease in the Chinese Population,” Aging (Albany NY) 13, no. 8 (2021): 11352–11362. 33833133 10.18632/aging.202827 PMC8109119

[44] M. Xiao , J. Xu , W. Wang , et al., “Functional Significance of Cholesterol Metabolism in Cancer: From Threat to Treatment,” Experimental & Molecular Medicine 55 (2023): 1982–1995. 37653037 10.1038/s12276-023-01079-w PMC10545798

[45] Y. Duan , K. Gong , S. Xu , F. Zhang , X. Meng , and J. Han , “Regulation of Cholesterol Homeostasis in Health and Diseases: From Mechanisms to Targeted Therapeutics,” Signal Transduction and Targeted Therapy 7 (2022): 265. 35918332 10.1038/s41392-022-01125-5 PMC9344793

[46] H. M. Jones , Z. Fang , W. Sun , et al., “Atorvastatin Exhibits Anti‐Tumorigenic and Anti‐Metastatic Effects in Ovarian Cancer in Vitro,” American Journal of Cancer Research 7, no. 12 (2017): 2478–2490. 29312801 PMC5752688

[47] R. Marques , C. J. Maia , C. Vaz , S. Correia , and S. Socorro , “The Diverse Roles of Calcium‐Binding Protein Regucalcin in Cell Biology: From Tissue Expression and Signaling to Disease,” Cellular and Molecular Life Sciences 71, no. 1 (2014): 93–111. 23519827 10.1007/s00018-013-1323-3 PMC11113322

[48] A. Zinellu , S. Sotgia , G. Loriga , L. Deiana , A. E. Satta , and C. Carru , “Oxidative Stress Improvement Is Associated With Increased Levels of Taurine in CKD Patients Undergoing Lipid‐Lowering Therapy,” Amino Acids 43, no. 4 (2012): 1499–1507 Effects of Ezetimibe/Simvastatin and Rosuvastatin on Oxidative Stress in Diabetic Neuropathy: A Randomized, Double‐Blind, Placebo‐Controlled Clinical Trial. Oxid Med Cell Longev. 2015:2015:756294. 22278741 10.1007/s00726-012-1223-0

[49] G. Villegas‐Rivera , L. M. Román‐Pintos , E. G. Cardona‐Muñoz , et al., “Effects of Ezetimibe/Simvastatin and Rosuvastatin on Oxidative Stress in Diabetic Neuropathy: A Randomized, Double‐Blind, Placebo‐Controlled Clinical Trial,” Oxidative Medicine and Cellular Longevity 2015 (2015): 756294. 26290682 10.1155/2015/756294 PMC4531178

[50] M. Valentovic , “Simvastatin,” in xPharm: The Comprehensive Pharmacology Reference, ed. S. J. Enna and D. B. Bylund (Elsevier, 2007), 1–4.

