# Supplementary material for: Deciphering the Mechanisms of Statin–Ezetimibe Drug Combinations Using Boolean Logical Modeling and Transcriptomic Data
Source: CPT Pharmacometrics Syst Pharmacol. 2026 Sep 1;15(9):e70329. doi: 10.1002/psp4.70329 (PMC13532064; doi:10.1002/psp4.70329)
Supplement: Supplementary file 1 [file PSP4-15-e70329-s001.docx]

**Deciphering the Mechanisms of Statin–Ezetimibe Drug Combinations Using Boolean Logical Modeling and Transcriptomic Data**

**Supplemental information**

**Drug-induced transcriptome datasets**

The first drug-induced RNA-seq count dataset was that from human SOAT2-only-HepG2 cells - a unique human hepatocyte cell model better simulating lipid and lipoprotein metabolism [20] (culture in human sera as described in [21] and treated for 16 hours with atorvastatin 5 umol/L, ezetimibe 25 umol/L, their combination, or vehicle). There were 6 replicates for each treatment, and, in total, there were 24 samples and 63,568 genomic features in this dataset. The detailed information about this dataset can be found in [22]. Table S1 and Figure S1 (Supplementary File 1) shows strong within-group sample correlations across all genes in SOAT2-only HepG2 cells under four treatment conditions, indicating the high-quality of the transcriptome data. We removed genome features that have less than 25 reading counts in all the samples. After that filtering step, 14,599 were left for further analysis. To ensure that the genes we investigated had meaningful changes, we used ANOVA to identify genes differentially expressed across the four conditions. Of these, there were 1,560 with p-values <0.01 after the Benjamini-Hochberg multiple testing correction.

Another drug-induced RNA-seq count dataset we utilized is that from liver biopsies of non-obese normolipidemic patients with uncomplicated cholesterol gallstone disease in the Stockholm Study (a single-blind, randomized trial) who received simvastatin 80 mg daily, ezetimibe 10 mg daily, alone or in combination, or placebo for four weeks prior to sample collection and liver biopsy [22]. The clinical trial was approved by the Stockholm Region Ethical Board (2006/1204-31/1) and the Swedish Medical Agency and registered in the EU clinical trial register (EudraCT number: 2006-004839-30). All patients gave written informed consent to participate in the study.  In this dataset, there are 8 placebo samples, 5 simvastatin treated samples, 5 ezetimibe treated samples, and 9 simvastatin-ezetimibe combination treated samples. In total, there were 27 samples and 58,381 genomic features. Within-group sample correlation and principal component analysis (PCA) showed that one simvastatin sample and two simvastatin+ezetimibe samples were extreme outliers (Table S3-S4, Figure S1, Supplementary File 1), for which reason we removed these samples from further analysis. The PCA map also shows that the patient data are more heterogeneous than the cell data. After removing genome features that have less than 25 reading counts in all the samples, 19,007 were left for further analysis. We used ANOVA to identify genes that were differentially expressed across the four conditions and identified 565 such genes with p-values <0.05.

**Boolean models for the combinatory actions of drugs**

We have previously applied a Boolean framework to model the transcriptome data derived from G-protein knockout microarrays, revealing a novel mode of heterotrimeric G-protein function [23]. Here, we adapted this approach to model the combinatorial modes of action of drug combinations. We defined three binary variables: ATO= 1 indicating atorvastatin treatment, ATO=0 denoting no atorvastatin treatment; SIM=1 indicating simvastatin treatment, and SIM=0 denoting no simvastatin treatment; and EZE =1 indicating ezetimibe treatment and EZE=0 denoting no ezetimibe treatment. The full set of 16 Boolean functions defined by the two variables is summarized in Supplemental Table S5 (Supplementary File 1) to describe the combinatorial modes of the statins and ezetimibe. For example, if the activation of some genes needs both atorvastatin and ezetimibe, these genes will be assigned to the mode described by a model B_2_(ATO, EZE)= ATO AND EZE (Figure 1A). If some genes can be induced by either atorvastatin or ezetimibe, these genes will be assigned to the mode described by a model B_8_(ATO, EZE)= ATO OR EZE. If a gene is inhibited by atorvastatin independently, it will be assigned to the mode described a model B_13_(ATO, EZE)= NOT ATO. Note that this framework models the coordinated (synergism) actions of ATO and ETE, but does not directly model their antagonistic actions, based on our knowledge of the beneficial effects of each of these agents. Even though we use Boolean models, our method does not need to truncate gene expression profiles into binary values. Instead, we use Boolean models as idealized expression templates (Figure 1B).

**Idealized differential expression templates**

To assign differentially expressed genes to the different combinatorial modes of action of the drug combination, we define idealized differential expression templates derived from the 16 Boolean models (Figure 1C). B_1_ and B_16_ represent the expression profiles of genes that do not change over different treatments and that have been filtered out during the ANOVA analysis. The remaining 14 Boolean models each defines an idealized differential expression template through pairwise comparisons of two treatment conditions (Table S5, Supplementary File 1). For example, if a gene is regulated by the action B_2_(ATO, EZE)=ATO AND EZE, its idealized expression is [0, 0, 0, 1] in the order of VC-ATO-EZE-DC, where DC represents ‘drug combination’. This expression pattern indicates that compared with expression in vehicle control (VC), the gene should be not differentially expressed in ATO, not differentially expressed in EZE, but up-regulated in the drug combination (DC); compared with ATO, the gene should have no differential expression in EZE and should be up-regulated in DC; and compared with EZE, the gene should be up-regulated in DC. Therefore, we have the differential expression pattern (VC~ATO, VC~EZE, VC<DC, ATO~EZE, ATO<DC, EZE<DC) elucidated by a total of six pairwise comparisons. If we let 1 represent up-regulation, -1 represent down-regulation, and 0 denote no differential expression, the gene regulated by the mode B_2_ should have an idealized differential expression pattern [0, 0, 1, 0, 1, 1] in the order VC vs. ATO, VC vs. EZE, VC vs. DC, ATO vs. EZE, ATO vs. DC, and EZE vs. DC.

**Pattern matching**

In order to associate differentially expressed genes with different regulatory action modes of the drug combination, we define an association metric between the differential expression pattern D*i*=(logFC*_i,_*_1_, logFC*_i,_*_2_,…, logFC*_i,6_*) of a gene *i* with the three-valued differential expression template *T*=[*t*_1_, *t*_2_,…, *t*_6_] of a Boolean function B(ATO, EZE) as follows:

Where *I* is an indicator function. The association metric gains a high score when a template entry is not zero, the fold change is large, and the change direction is consistent with the template entry. The association metric penalizes the score when the template entry is zero and the fold change is large, or when the template entry is not zero, but the direction of the fold change is opposite that of the template entry. Each gene has 14 association scores representing the matching extent of the genes with the 14 regulatory modes of the drug combination. Through this analysis, we can know what types of combinatorial regulation modes are prevalent for the statins and ezetimibe. The pattern matching approach takes the original differential expression profiles as input instead of truncating differential gene expression patterns into trinary values.

**Human protein-protein interactome**

In our previous research, we have curated a high-quality human protein-protein interactome consisting of 16,454 proteins and 233,957 physical protein-protein interactions (PPIs) directly from experimental high-throughput interactome studies [24]. The most recent reference map of the human binary protein interactome (HuRI) with approximately 53,000 interactions has been included in this interactome[25]. The human interactome also included high-quality PPIs profiled via affinity-purification mass spectrometry, such as the BioPlex method. In addition, we have also compiled signaling interactions and kinase-substrate interactions from literature and unbiased databases. Detailed descriptions of the construction of this human interactome can be found in our prior publication [24]. In this study, we update the human interactome by including Bioplex 3.0 which consists of nearly 120,000 interactions among nearly 15,000 proteins and is the most comprehensive experimentally derived model of the human interactome to date [26]. This inclusion leads to the latest version of the human interactome consisting of 17,350 proteins and 350,950 interactions.

**Pathway and disease enrichment analysis**

To examine whether specific combinatorial modes of the statins and ezetimibe tend to be involved in specific signaling pathways, we performed functional analysis on the genes assigned in prevalent combinatorial modes using MetaScape, a gene list analysis tool that integrates over 40 function and pathway knowledgebases, including GO Biological Processes, Reactome, and KEGG Pathway[33]. The p-values are calculated using the hypergeometric cumulative distribution, and the Benjamini-Hochberg procedure was applied for multiple testing correction. To understand the additional indications and therapeutic mechanisms of the atorvastatin/simvastatin and ezetimibe combination, we also examined whether the subnetworks induced by the drug combinations are significantly enriched with disease-related genes using MetaScape based on the disease-gene associations from DisGeNet.

Table S1. Within-group sample correlation over all genes in SOAT2-only-HepG2 cells under four treatment conditions. VC: Vehicle control; ATO: atorvastatin; EZE: ezetimibe.

|  | VC2 | VC3 | VC4 | VC5 | VC6 |
| --- | --- | --- | --- | --- | --- |
| VC1 | 0.996 | 0.996 | 0.990 | 0.995 | 0.992 |
| VC2 |  | 0.997 | 0.996 | 0.995 | 0.989 |
| VC3 |  |  | 0.998 | 0.999 | 0.990 |
| VC4 |  |  |  | 0.997 | 0.986 |
| VC5 |  |  |  |  | 0.992 |
|  | ATO2 | ATO3 | ATO4 | ATO5 | ATO6 |
| ATO1 | 0.991 | 0.953 | 0.989 | 0.995 | 0.950 |
| ATO2 |  | 0.968 | 0.989 | 0.986 | 0.954 |
| ATO3 |  |  | 0.976 | 0.938 | 0.912 |
| ATO4 |  |  |  | 0.981 | 0.935 |
| ATO5 |  |  |  |  | 0.943 |
|  | EZE2 | EZE3 | EZE4 | EZE5 | EZE6 |
| EZE1 | 0.944 | 0.902 | 0.936 | 0.953 | 0.956 |
| EZE2 |  | 0.988 | 0.960 | 0.994 | 0.890 |
| EZE3 |  |  | 0.962 | 0.971 | 0.837 |
| EZE4 |  |  |  | 0.939 | 0.821 |
| EZE5 |  |  |  |  | 0.923 |
|  | ATO+EZE2 | ATO+EZE3 | ATO+EZE4 | ATO+EZE5 | ATO+EZE6 |
| ATO+EZE1 | 0.971 | 0.905 | 0.966 | 0.980 | 0.972 |
| ATO+EZE2 |  | 0.941 | 0.994 | 0.994 | 0.999 |
| ATO+EZE3 |  |  | 0.933 | 0.952 | 0.947 |
| ATO+EZE4 |  |  |  | 0.993 | 0.994 |
| ATO+EZE5 |  |  |  |  | 0.996 |

Table S2. Within-group sample correlation over all genes in liver biopsies of patients given placebo treatment. PLA: placebo.

|  | PLA _15 | PLA_21 | PLA_23 | PLA_27 | PLA_32 | PLA_29 | PLA_38 |
| --- | --- | --- | --- | --- | --- | --- | --- |
| PLA_6 | 0.994 | 0.964 | 0.986 | 0.989 | 0.985 | 0.989 | 0.866 |
| PLA _15 |  | 0.960 | 0.995 | 0.989 | 0.994 | 0.994 | 0.863 |
| PLA_21 |  |  | 0.952 | 0.979 | 0.947 | 0.939 | 0.962 |
| PLA_23 |  |  |  | 0.981 | 0.998 | 0.990 | 0.851 |
| PLA_27 |  |  |  |  | 0.979 | 0.983 | 0.905 |
| PLA_32 |  |  |  |  |  | 0.990 | 0.843 |
| PLA_29 |  |  |  |  |  |  | 0.826 |

Table S3. Within-group sample correlation over all genes in liver biopsies of patients given drug treatments. SIM: simvastatin; EZE: ezetimibe.

|  | SIM_13b | SIM_26 | SIM_36 | SIM_37 |
| --- | --- | --- | --- | --- |
| SIM_1 | 0.965 | **0.582** | 0.967 | 0.928 |
| SIM_13b |  | **0.604** | 0.998 | 0.975 |
| SIM_26 |  |  | **0.595** | **0.430** |
| SIM_36 |  |  |  | 0.975 |
|  | EZE_5b | EZE_18 | EZE_24 | EZE_31A |
| EZE_4 | 0.944 | 0.982 | 0.946 | 0.906 |
| EZE_5b |  | 0.978 | 0.806 | 0.735 |
| EZE_18 |  |  | 0.894 | 0.826 |
| EZE_24 |  |  |  | 0.979 |

Table S4. Within-group sample correlation over all genes in liver biopsies of patients given the combination of simvastatin and ezetimibe treatment. SIM: simvastatin; EZE: ezetimibe.

|  | SIM+  EZE_11 | SIM+  EZE_14 | SIM+  EZE_16 | SIM+  EZE_28 | SIM+  EZE_30 | SIM+  EZE_33 | SIM+  EZE_35 | SIM+  EZE_39 |
| --- | --- | --- | --- | --- | --- | --- | --- | --- |
| SIM+EZE_7 | 0.834 | 0.995 | 0.964 | 0.948 | **0.501** | 0.989 | 0.986 | **0.275** |
| SIM+EZE_11 |  | 0.841 | 0.884 | 0.917 | **0.529** | 0.867 | 0.857 | **0.330** |
| SIM+EZE_14 |  |  | 0.968 | 0.951 | **0.486** | 0.989 | 0.985 | **0.268** |
| SIM+EZE_16 |  |  |  | 0.985 | **0.477** | 0.970 | 0.963 | **0.277** |
| SIM+EZE_28 |  |  |  |  | **0.451** | 0.952 | 0.962 | **0.364** |
| SIM+EZE_30 |  |  |  |  |  | **0.439** | **0.587** | 0.768 |
| SIM+EZE_33 |  |  |  |  |  |  |  | **0.224** |
| SIM+EZE_35 |  |  |  |  |  |  |  | **0.362** |

**Figure S1. PCA mappings of samples from human SOAT2-only-HepG2 cells and liver biopsies.** VC: Vehicle control; ATO: atorvastatin; EZE: ezetimibe. PLA: placebo. SIM: simvastatin; EZE: ezetimibe.

**Figure S2. Network mapping (A) of genes assigned to B_4_(ATO, EZE)=ATO and disease enrichment analysis (B)**.

Table S5. Boolean functions modeling the combinatorial modes of atorvastatin and ezetimibe and the idealized gene expression patterns. In the case of simvastatin and ezetimibe, ATO is replaced with SIM.

| **Action modes of drug combination** | **VC** | **ATO** | **EZE** | **DC** | **VC—ATO—EZE—DC** |
| --- | --- | --- | --- | --- | --- |
| B_1_(ATO, EZE)=0 | 0 | 0 | 0 | 0 |  |
| B_2_(ATO, EZE)=ATO AND EZE | 0 | 0 | 0 | 1 |  |
| B_3_(ATO, EZE)=ATO AND NOT EZE | 0 | 1 | 0 | 0 |  |
| B_4_(ATO, EZE)=ATO | 0 | 1 | 0 | 1 |  |
| B_5_(ATO, EZE)=NOT ATO AND EZE | 0 | 0 | 1 | 0 |  |
| B_6_(ATO, EZE)=EZE | 0 | 0 | 1 | 1 |  |
| B_7_(ATO, EZE)=(ATO OR EZE) AND NOT (ATO AND EZE) | 0 | 1 | 1 | 0 |  |
| B_8_(ATO, EZE)=ATO OR EZE | 0 | 1 | 1 | 1 |  |
| B_9_(ATO, EZE)=NOT (ATO AND EZE) | 1 | 0 | 0 | 0 |  |
| B_10_(ATO, EZE)=NOT (ATO OR EZE) OR (ATO AND EZE) | 1 | 0 | 0 | 1 |  |
| B_11_(ATO, EZE)=NOT EZE | 1 | 1 | 0 | 0 |  |
| B_12_(ATO, EZE)=ATO OR NOT EZE | 1 | 1 | 0 | 1 |  |
| B_13_(ATO, EZE)=NOT ATO | 1 | 0 | 1 | 0 |  |
| B_14_(ATO, EZE)=NOT ATO OR EZE | 1 | 0 | 1 | 1 |  |
| B_15_(ATO, EZE)=NOT (ATO AND EZE) | 1 | 1 | 1 | 0 |  |
| B_16_(ATO, EZE)=1 | 1 | 1 | 1 | 1 |  |

Table S6. Boolean functions modeling the combinatorial modes of atorvastatin and ezetimibe. In the case of simvastatin and ezetimibe, ATO is replaced with SIM, and VC is replaced with PLB (placebo).

|  | ATO~VC | EZE~VC | DC~VC | EZE~ATO | DC~ATO | EZE~ATO | Idealized differential expression templates |
| --- | --- | --- | --- | --- | --- | --- | --- |
| B_2_ | 0 | 0 | 1 | 0 | 1 | 1 |  |
| B_3_ | 1 | 0 | 0 | -1 | -1 | 0 |  |
| B_4_ | 1 | 0 | 1 | -1 | 0 | 1 |  |
| B_5_ | 0 | 1 | 0 | 1 | 0 | -1 |  |
| B_6_ | 0 | 1 | 1 | 1 | 1 | 0 |  |
| B_7_ | 1 | 1 | 0 | 0 | -1 | -1 |  |
| B_8_ | 1 | 1 | 1 | 0 | 0 | 0 |  |
| B_9_ | -1 | -1 | -1 | 0 | 0 | 0 |  |
| B_10_ | -1 | -1 | 0 | 0 | 1 | 1 |  |
| B_11_ | 0 | -1 | -1 | -1 | -1 | 0 |  |
| B_12_ | 0 | -1 | 0 | -1 | 0 | 1 |  |
| B_13_ | -1 | 0 | -1 | 1 | 0 | -1 |  |
| B_14_ | -1 | 0 | 0 | 1 | 1 | 0 |  |
| B_15_ | 0 | 0 | -1 | 0 | -1 | -1 |  |
